# Supplementary material for: Modeling glioblastoma heterogeneity as a dynamic network of cell states
Source: Mol Syst Biol. 2021 Sep 16;17(9):e10105. doi: 10.15252/msb.202010105 (PMC8444284; doi:10.15252/msb.202010105)
Supplement: Supplementary file 6 — Source Data for Figure 5 [file MSB-17-e10105-s004.zip › Figure5A_sourcedata/GSEA_3017/nosigdb_stateE.GseaPreranked.1622795031093/RICHARDS_ZHONG_NATURE_2018_ASTROCYTES_UPREG.html]

Details for gene set RICHARDS\_ZHONG\_NATURE\_2018\_ASTROCYTES\_UPREG[GSEA]

|  || Dataset | state63017 |
| Phenotype | NoPhenotypeAvailable |
| Upregulated in class | na\_pos |
| GeneSet | RICHARDS\_ZHONG\_NATURE\_2018\_ASTROCYTES\_UPREG |
| Enrichment Score (ES) | 0.5925148 |
| Normalized Enrichment Score (NES) | 4.0195494 |
| Nominal p-value | 0.0 |
| FDR q-value | 0.0 |
| FWER p-Value | 0.0 |
Table: GSEA Results Summary

  

Fig 1: Enrichment plot: RICHARDS\_ZHONG\_NATURE\_2018\_ASTROCYTES\_UPREG      
 Profile of the Running ES Score & Positions of GeneSet Members on the Rank Ordered List

  

| PROBE | GENE SYMBOL | GENE\_TITLE | RANK IN GENE LIST | RANK METRIC SCORE | RUNNING ES | CORE ENRICHMENT || 1 | PMP22 |  |  | 0 | 1.393 | 0.0481 | Yes |
| 2 | GFAP |  |  | 1 | 1.383 | 0.0959 | Yes |
| 3 | SPARCL1 |  |  | 7 | 0.963 | 0.1236 | Yes |
| 4 | LRRN3 |  |  | 10 | 0.943 | 0.1539 | Yes |
| 5 | FABP7 |  |  | 11 | 0.920 | 0.1857 | Yes |
| 6 | TTYH1 |  |  | 19 | 0.731 | 0.2031 | Yes |
| 7 | CNN3 |  |  | 24 | 0.702 | 0.2228 | Yes |
| 8 | LGALS3 |  |  | 25 | 0.697 | 0.2469 | Yes |
| 9 | IFITM3 |  |  | 26 | 0.682 | 0.2705 | Yes |
| 10 | MSMO1 |  |  | 28 | 0.648 | 0.2918 | Yes |
| 11 | CYP51A1 |  |  | 30 | 0.637 | 0.3126 | Yes |
| 12 | GJA1 |  |  | 33 | 0.618 | 0.3318 | Yes |
| 13 | RAB31 |  |  | 35 | 0.598 | 0.3513 | Yes |
| 14 | HMGCS1 |  |  | 37 | 0.593 | 0.3707 | Yes |
| 15 | C1orf61 |  |  | 43 | 0.553 | 0.3841 | Yes |
| 16 | ID1 |  |  | 45 | 0.542 | 0.4017 | Yes |
| 17 | UBL3 |  |  | 53 | 0.514 | 0.4116 | Yes |
| 18 | F3 |  |  | 55 | 0.506 | 0.4280 | Yes |
| 19 | MRC2 |  |  | 56 | 0.505 | 0.4454 | Yes |
| 20 | SLC1A3 |  |  | 66 | 0.472 | 0.4516 | Yes |
| 21 | SERPINB6 |  |  | 77 | 0.440 | 0.4555 | Yes |
| 22 | PDPN |  |  | 79 | 0.438 | 0.4695 | Yes |
| 23 | DOK5 |  |  | 81 | 0.437 | 0.4835 | Yes |
| 24 | CST3 |  |  | 86 | 0.425 | 0.4937 | Yes |
| 25 | GPM6B |  |  | 105 | 0.391 | 0.4869 | Yes |
| 26 | TIMP3 |  |  | 106 | 0.391 | 0.5004 | Yes |
| 27 | LGALS1 |  |  | 113 | 0.368 | 0.5063 | Yes |
| 28 | CLU |  |  | 119 | 0.364 | 0.5133 | Yes |
| 29 | SEMA6A |  |  | 130 | 0.348 | 0.5140 | Yes |
| 30 | GATM |  |  | 149 | 0.335 | 0.5053 | Yes |
| 31 | ZFP36L2 |  |  | 162 | 0.324 | 0.5030 | Yes |
| 32 | DDAH1 |  |  | 170 | 0.317 | 0.5060 | Yes |
| 33 | JUN |  |  | 172 | 0.316 | 0.5158 | Yes |
| 34 | FADS2 |  |  | 181 | 0.310 | 0.5175 | Yes |
| 35 | LRRC17 |  |  | 182 | 0.310 | 0.5282 | Yes |
| 36 | PLIN3 |  |  | 192 | 0.301 | 0.5285 | Yes |
| 37 | SLC25A18 |  |  | 195 | 0.300 | 0.5366 | Yes |
| 38 | GLUL |  |  | 196 | 0.300 | 0.5469 | Yes |
| 39 | SDC3 |  |  | 200 | 0.295 | 0.5537 | Yes |
| 40 | DKK3 |  |  | 205 | 0.293 | 0.5593 | Yes |
| 41 | GNG11 |  |  | 210 | 0.289 | 0.5648 | Yes |
| 42 | GNG12 |  |  | 212 | 0.287 | 0.5736 | Yes |
| 43 | FERMT2 |  |  | 219 | 0.283 | 0.5766 | Yes |
| 44 | PTTG1IP |  |  | 225 | 0.280 | 0.5807 | Yes |
| 45 | DAG1 |  |  | 228 | 0.279 | 0.5881 | Yes |
| 46 | QKI |  |  | 241 | 0.267 | 0.5838 | Yes |
| 47 | SLC4A4 |  |  | 258 | 0.259 | 0.5747 | Yes |
| 48 | LEPROT |  |  | 259 | 0.259 | 0.5836 | Yes |
| 49 | ATP1B2 |  |  | 260 | 0.258 | 0.5925 | Yes |
| 50 | ZFP36L1 |  |  | 287 | -0.255 | 0.5720 | No |
| 51 | CA12 |  |  | 325 | -0.263 | 0.5394 | No |
| 52 | BCHE |  |  | 471 | -0.305 | 0.3865 | No |
| 53 | CSPG5 |  |  | 535 | -0.328 | 0.3268 | No |
| 54 | ETV1 |  |  | 563 | -0.341 | 0.3081 | No |
| 55 | SERPINE2 |  |  | 585 | -0.351 | 0.2966 | No |
| 56 | DNER |  |  | 640 | -0.382 | 0.2489 | No |
| 57 | SEZ6L |  |  | 642 | -0.383 | 0.2610 | No |
| 58 | PSAT1 |  |  | 720 | -0.448 | 0.1897 | No |
| 59 | DBI |  |  | 776 | -0.526 | 0.1458 | No |
| 60 | FABP5 |  |  | 939 | -1.294 | 0.0079 | No |
Table: GSEA details [plain text format]

  

Fig 2: RICHARDS\_ZHONG\_NATURE\_2018\_ASTROCYTES\_UPREG: Random ES distribution      
 Gene set null distribution of ES for **RICHARDS\_ZHONG\_NATURE\_2018\_ASTROCYTES\_UPREG**
